# LINC01607 promotes hepatocellular carcinoma progression and ferroptosis-associated therapy resistance with functional involvement of the p62–Keap1–Nrf2 pathway

**DOI:** 10.20517/cdr.2026.02

**Published:** 2026-07-17

**Authors:** Yuxin Zhang, Weiqi Xu, Fangling Cheng, Jinghan Zhu, Yuanxiang Lu, Guangzhen Cai, Jiang Li, Yujie Zhang

**Affiliations:** ^1^Hepatic Surgery Center, Tongji Hospital, Tongji Medical College, Huazhong University of Science and Technology, Wuhan 430030, Hubei, China.; ^2^Department of Obstetrics and Gynecology, National Clinical Research Center for Obstetrics and Gynecology, Tongji Hospital, Tongji Medical College, Huazhong University of Science and Technology, Wuhan 430030, Hubei, China.; ^3^Department of General Surgery, The First Affiliated Hospital of Nanchang University, Nanchang 330000, Jiangxi, China.; ^4^Department of Oncology, Tongji Hospital, Tongji Medical College, Huazhong University of Science and Technology, Wuhan 430030, Hubei, China.; ^5^Department of Hepatobiliary and Pancreatic Surgery, First Affiliated Hospital, College of Medicine, Shihezi University, Shihezi 832008, Xinjiang, China.; ^#^Authors contributed equally to this work.

**Keywords:** Hepatocellular carcinoma, LINC01607, ferroptosis, sorafenib resistance

## Abstract

**Aim:** Hepatocellular carcinoma (HCC) remains a formidable worldwide health challenge, characterized by inadequate treatment efficacy and unsatisfactory clinical prognosis. Our previous study implicated LINC01607 in lenvatinib resistance, but its role in HCC progression and ferroptosis-associated vulnerability remains unclear.

**Methods:** LINC01607 expression was examined in HCC patient samples and The Cancer Genome Atlas datasets. Cellular, animal, and patient-derived organoid (PDO) models were used to evaluate its biological function. RNA sequencing, ferroptosis-related assays, rescue experiments, and drug-sensitivity analyses were performed to explore associated downstream pathways.

**Results:** LINC01607 was upregulated in HCC tissues and associated with aggressive clinicopathological features and poor survival. Functional assays showed that LINC01607 promoted HCC cell proliferation, migration, invasion, tumor growth, and metastasis. LINC01607 depletion induced ferroptosis-associated changes, including increased lipid peroxidation, glutathione depletion, and Fe^2+^ accumulation under ferroptotic stress, which were partially reversed by ferroptosis inhibitors. LINC01607 knockdown also enhanced sensitivity to RSL3 and sorafenib, while ferrostatin-1 partially rescued the increased sorafenib sensitivity. RNA sequencing and rescue experiments suggested involvement of the p62–Keap1–Nrf2 pathway. LINC01607 depletion was associated with reduced SQSTM1/p62, Nrf2, and ferroptosis-resistance proteins, whereas p62 overexpression partially reversed these effects and Nrf2 knockdown abrogated the rescue. In xenograft and PDO models, LINC01607 depletion improved the response to sorafenib.

**Conclusion:** LINC01607 contributes to HCC progression and ferroptosis-associated therapy resistance, at least in part through the p62–Keap1–Nrf2 pathway, supporting further investigation of LINC01607 as a potential therapeutic target.

## INTRODUCTION

Hepatocellular carcinoma (HCC) is a major oncological challenge worldwide, ranking sixth in new cancer cases and third in cancer mortality^[1]^. Because HCC typically progresses aggressively while producing few early symptoms, many patients present with advanced disease, at which point curative resection is usually no longer feasible, and clinical outcomes remain poor. Despite the approval of multikinase inhibitors, such as sorafenib and lenvatinib, by the US Food and Drug Administration (FDA), these treatments extend survival by only two to three months, and the median survival for advanced-stage patients is less than one year^[2,3]^. Insufficient treatment efficacy and drug resistance remain major obstacles in HCC management, emphasizing the importance of uncovering the molecular mechanisms that drive tumor progression and resistance.

Long noncoding RNAs (lncRNAs) are increasingly recognized as important modulators of cellular activity, regulating gene expression and shaping diverse biological functions^[4,5]^. Among these, specific lncRNAs have been implicated as either oncogenes or tumor suppressors. Accumulating evidence indicates that lncRNAs are closely involved in HCC progression. These HCC-related lncRNAs can influence various cancerous characteristics by altering gene expression or protein activity. A more comprehensive understanding of the lncRNA regulatory network involved in the multistage process of HCC development will shed new light on the diagnosis and treatment of HCC^[6,7]^. Our previous work in lenvatinib-resistant HCC revealed that LINC01607 is a predominantly cytoplasmic lncRNA that can upregulate p62 through a miR-892b-dependent ceRNA mechanism^[8]^. Building on that observation, the present study investigated whether LINC01607 also promotes HCC progression and ferroptosis resistance through downstream functional modulation of the p62–Keap1–NRF2 pathway.

Ferroptosis is distinguished from other forms of cell death by its dependence on iron and the accumulation of lipid peroxides, making it a promising focus in cancer therapy research^[9,10]^. This process is closely linked to GPX4 suppression, disruption of the glutathione (GSH) antioxidant system, iron accumulation, and lipid reactive oxygen species (ROS) generation, often following inhibition of the cystine/glutamate transporter SLC7A11/xCT. When the accumulation of intracellular ROS exceeds the capacity of GSH and GPX4, lipid peroxidation occurs, triggering ferroptosis. Agents such as GPX4 inhibitors (e.g., RSL3) and compounds such as erastin and sorafenib, which target xCT-mediated cystine import, have been shown to be effective at inducing ferroptosis^[11,12]^. While triggering ferroptosis is a new approach to curb HCC, malignant cells can develop adaptive mechanisms that lead to resistance against ferroptosis and related drugs^[13,14]^. In this context, defining ferroptosis-related adaptive mechanisms in HCC may help identify therapeutic strategies to increase tumor vulnerability and improve drug response. Therefore, targeting LINC01607 may represent a potential strategy to enhance ferroptosis-associated vulnerability and improve sorafenib response.

Oxidative stress, resulting from disrupted redox homeostasis between ROS and antioxidant defenses, is a critical factor involved in liver tumorigenesis^[15]^. In the ferroptosis process, excessive ROS-driven lipid peroxidation is the key event. HCC cells have evolved a robust antioxidant system to counteract ROS-induced oxidative damage^[16]^. Nrf2, a master regulator of cellular defense against oxidative stress, orchestrates the expression of detoxifying and antioxidant proteins^[17,18]^. Normally, Keap1 facilitates the ubiquitin-proteasome-mediated degradation of Nrf2. Upon oxidative stress, modification of cysteine residues in Keap1 disrupts this process, enabling Nrf2 nuclear accumulation and the transcriptional activation of cytoprotective genes. The stress-responsive protein SQSTM1/p62 interacts with Keap1, stabilizing Nrf2 and amplifying its activity^[19,20]^. The p62–Keap1–Nrf2 axis has been implicated in oxidative stress management, ferroptosis regulation, and tumor resistance to treatment^[21,22]^. Therefore, this axis may contribute to HCC progression and treatment resistance. In this study, we found that LINC01607 promotes HCC proliferation and metastasis and contributes to ferroptosis-associated resistance, with functional involvement of the p62–Keap1–Nrf2 pathway.

## METHODS

### Clinical samples

Our study utilized two sets of HCC samples. The first set comprised 92 paired HCC tissues, which were analyzed for LINC01607 expression, and the patient characteristics of the LINC01607-high and LINC01607-low groups are detailed in Table 1. The second set included freshly collected surgical specimens used to establish organoid cultures for *in vitro* assessment of various interventions. Six independent HCC patient-derived organoid (PDO) lines were successfully generated and included in the present study. Baseline LINC01607 expression was measured in each PDO line by quantitative reverse transcription polymerase chain reaction (qRT-PCR) before the intervention experiments. All procedures involving human participants were approved by the Ethics Committee of Tongji Hospital (approval No. TJ-IRB20230863) and were conducted in accordance with the Declaration of Helsinki. For the retrospective analysis of archived clinical specimens and de-identified clinical data, a waiver of informed consent was granted by the ethics committee. For prospectively collected fresh clinical specimens used to establish PDO models, written informed consent was obtained from the corresponding patients. All animal experiments were approved by the Institutional Ethics Committee of Tongji Hospital (approval No. TJH-202212001) and were conducted in accordance with institutional animal welfare and ethical guidelines.

**Table 1:** Associations between LINC01607 and clinicopathological parameters in HCC

**Variables**	**Low-LINC01607 (*N* = 52)**	**High-LINC01607 (*N* = 40)**	***P* value**
Age, years, ≥ 50/< 50	42/10	28/12	0.17
Sex, male/female	25/27	18/22	0.467
ALT, U/L, < 40/≥ 40	27/25	21/19	0.562
AST, U/L, < 40/≥ 40	27/25	16/24	0.177
Liver cirrhosis, yes/no	29/23	21/19	0.46
HBsAg, negative/positive	25/27	17/23	0.374
AFP, ng/mL, < 55/≥ 55	5/47	6/34	0.319
Encapsulation, absent/present	28/24	19/21	0.347
Tumor size, cm, < 5/≥ 5	26/26	21/19	0.489
Tumor number, single/multiple	34/18	15/25	0.007
BCLC stage, A/B-C	47/5	34/6	0.319
Tumor differentiation, moderate/poor	37/15	17/23	0.05
Micro-vascular invasion, yes/no	17/35	22/18	0.026
TNM, I-II/III-IV	43/9	14/26	< 0.01

HCC: Hepatocellular carcinoma; ALT: alanine aminotransferase; AST: aspartate aminotransferase; AFP: alpha-fetoprotein; BCLC: Barcelona Clinic Liver Cancer; TNM: Tumor–Node–Metastasis.

### Cell culture, transfection, and molecular assays

Hep3B and Huh7 cells were used for *in vitro* functional and mechanistic experiments. LINC01607 knockdown or overexpression, SQSTM1/p62 overexpression, and Nrf2 knockdown were performed as indicated. qRT-PCR, fluorescence *in situ* hybridization (FISH), western blotting, lipid ROS measurement, C11-BODIPY staining, intracellular Fe^2+^ measurement, and GSH assays were conducted to evaluate LINC01607 expression, pathway alterations, and ferroptosis-associated changes. Detailed experimental procedures, reagent information, and siRNA/shRNA sequences are provided in Supplementary Methods and Supplementary Tables 1 and 2.

### Cell proliferation, migration, invasion, and drug-sensitivity assays

Cell Counting Kit-8 (CCK-8), 5-ethynyl-2′-deoxyuridine (EdU) incorporation, colony formation, Transwell, and wound-healing assays were performed to evaluate cell proliferation, migration, and invasion. Drug-sensitivity assays were used to assess the effects of LINC01607 modulation on responses to sorafenib, RSL3, and ferroptosis inhibitors. Detailed procedures are described in Supplementary Methods.

### Xenograft and metastasis models

Male BALB/c nude mice were used to evaluate tumor growth and metastatic potential *in vivo*. Hep3B cells with LINC01607 overexpression, knockdown, or corresponding controls were used to establish subcutaneous, orthotopic, and metastasis models as indicated. Tumor growth, tumor weight, bioluminescence signals, and histological changes were analyzed. All animal experiments were approved by the Institutional Ethics Committee of Tongji Hospital and performed in accordance with the ARRIVE guidelines (TJH-202212001). Detailed procedures are provided in Supplementary Methods.

### PDO models

Fresh HCC tissues were used to establish PDO models. Organoid identity was evaluated by histological and immunohistochemical analyses, and organoid viability was assessed after LINC01607 silencing, sorafenib treatment, or combined intervention. Detailed culture conditions and medium components are provided in Supplementary Methods and Supplementary Table 3.

### RNA sequencing

Total RNA was isolated from Hep3B cells transfected with siLINC01607 or negative control siRNA. Three independent biological replicates were included in each group. RNA sequencing and downstream bioinformatic analyses were performed to identify differentially expressed genes and enriched pathways. Detailed sequencing procedures are provided in Supplementary Methods.

### Statistical analysis

Statistical analyses were performed using SPSS 22.0. Data are presented as the mean ± standard deviation (SD). Comparisons between groups were performed using Student’s *t*-test, Mann–Whitney *U* test, chi-square test, or repeated-measures analysis of variance (ANOVA), as appropriate. Survival analyses were performed using Kaplan–Meier analysis, log-rank testing, and Cox proportional hazards regression models. Statistical significance was set at *P* < 0.05.

## RESULTS

### LINC01607 is upregulated in HCC tissues and is correlated with poor prognosis

**Figure 1:**
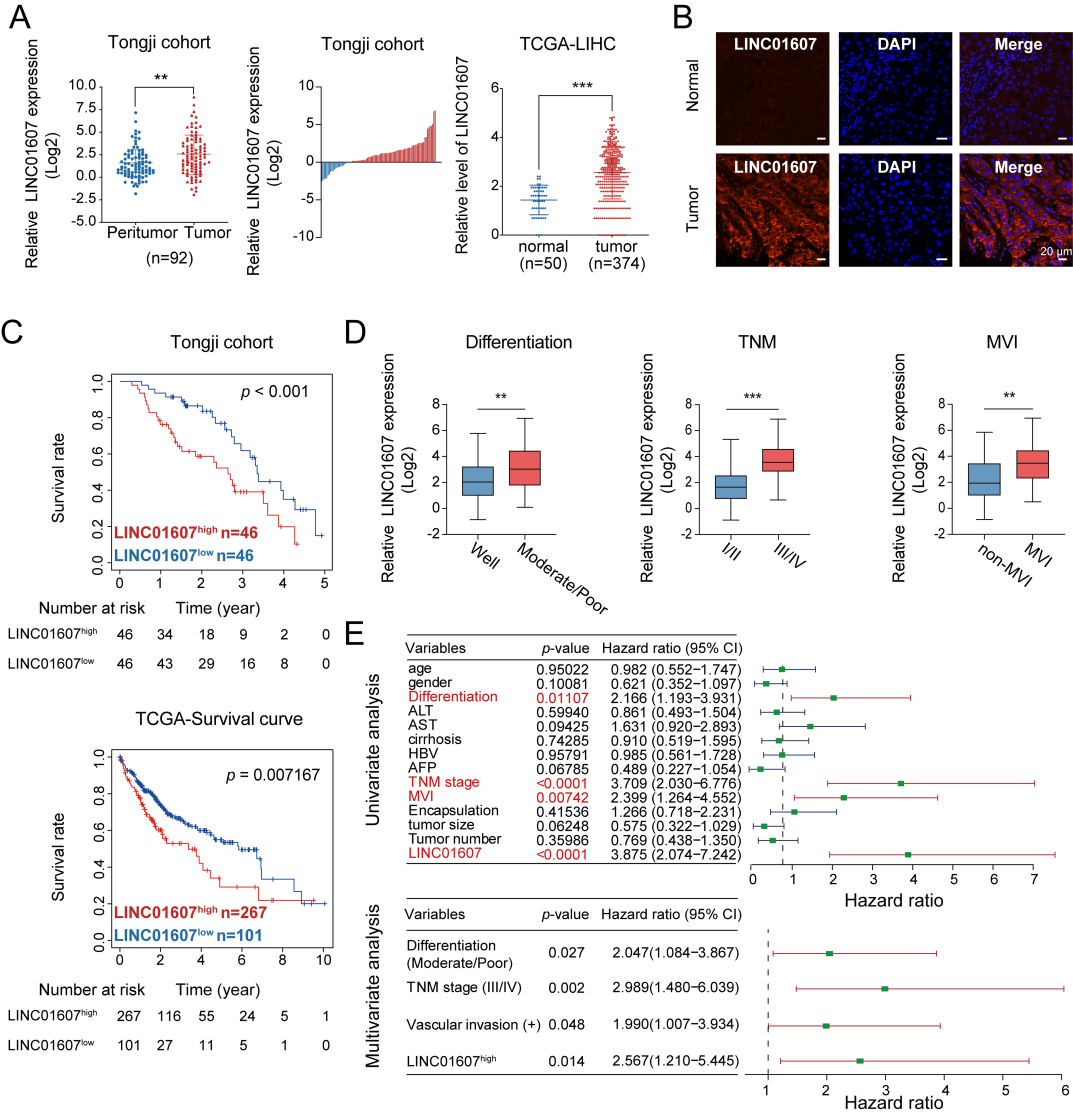
LINC01607 is upregulated in HCC and predicts unfavorable clinical outcomes. (A and B) LINC01607 expression levels in HCC tissues and corresponding peritumor liver tissues were assessed using qRT-PCR (A) and FISH (B). Both the Tongji cohort and TCGA-LIHC dataset showed higher LINC01607 expression in HCC tissues than in corresponding adjacent nontumor tissues; (C) Kaplan–Meier analysis indicated that patients with high LINC01607 expression had shorter overall survival in both the Tongji cohort and TCGA dataset; (D) The correlation between LINC01607 expression and tumor differentiation, TNM staging, and microvascular invasion in HCC patients; (E) Forest plots show univariate and multivariate Cox regression analyses of clinicopathological factors. LINC01607 expression, tumor differentiation, TNM stage, and vascular invasion were independent predictors of reduced overall survival. “n” indicates the number of patient samples. Survival differences were assessed using Kaplan–Meier analysis with the log-rank test. Cox proportional hazards regression was used for univariate and multivariate survival analyses. Other comparisons were performed using Student’s *t*-test, chi-square test, or Fisher’s exact test, as appropriate. ^**^P < 0.01, ^***^P < 0.001. HCC: Hepatocellular carcinoma; qRT-PCR: quantitative reverse transcription polymerase chain reaction; FISH: fluorescence *in situ* hybridization; TCGA-LIHC: The Cancer Genome Atlas Liver Hepatocellular Carcinoma; TNM: Tumor–Node–Metastasis; DAPI: 4′,6-diamidino-2-phenylindole; MVI: microvascular invasion; ALT: alanine aminotransferase; AST: aspartate aminotransferase; HBV: hepatitis B virus; AFP: alpha-fetoprotein.

Based on our previous gene expression profiling data, we further evaluated LINC01607 expression in paired HCC and adjacent peritumor liver tissues by qRT-PCR and FISH. LINC01607 levels were substantially higher in HCC specimens than in matched adjacent nontumor tissues. Elevated LINC01607 expression predicted shorter overall survival in both the Tongji cohort and The Cancer Genome Atlas Liver Hepatocellular Carcinoma (TCGA-LIHC) dataset [Figure 1A-C]. Further clinicopathological analysis showed that high LINC01607 expression was associated with poor differentiation, microvascular invasion, and advanced Tumor–Node–Metastasis (TNM) stage [Figure 1D]. Cox proportional hazards analysis further revealed that elevated LINC01607 levels, in addition to these clinical factors, were independent predictors of reduced overall survival [Figure 1E]. Overall, these results identify LINC01607 as an upregulated lncRNA in HCC that is associated with unfavorable clinicopathological and survival outcomes.

### LINC01607 promotes HCC cell proliferation and metastasis *in vitro* and *in vivo*

**Figure 2:**
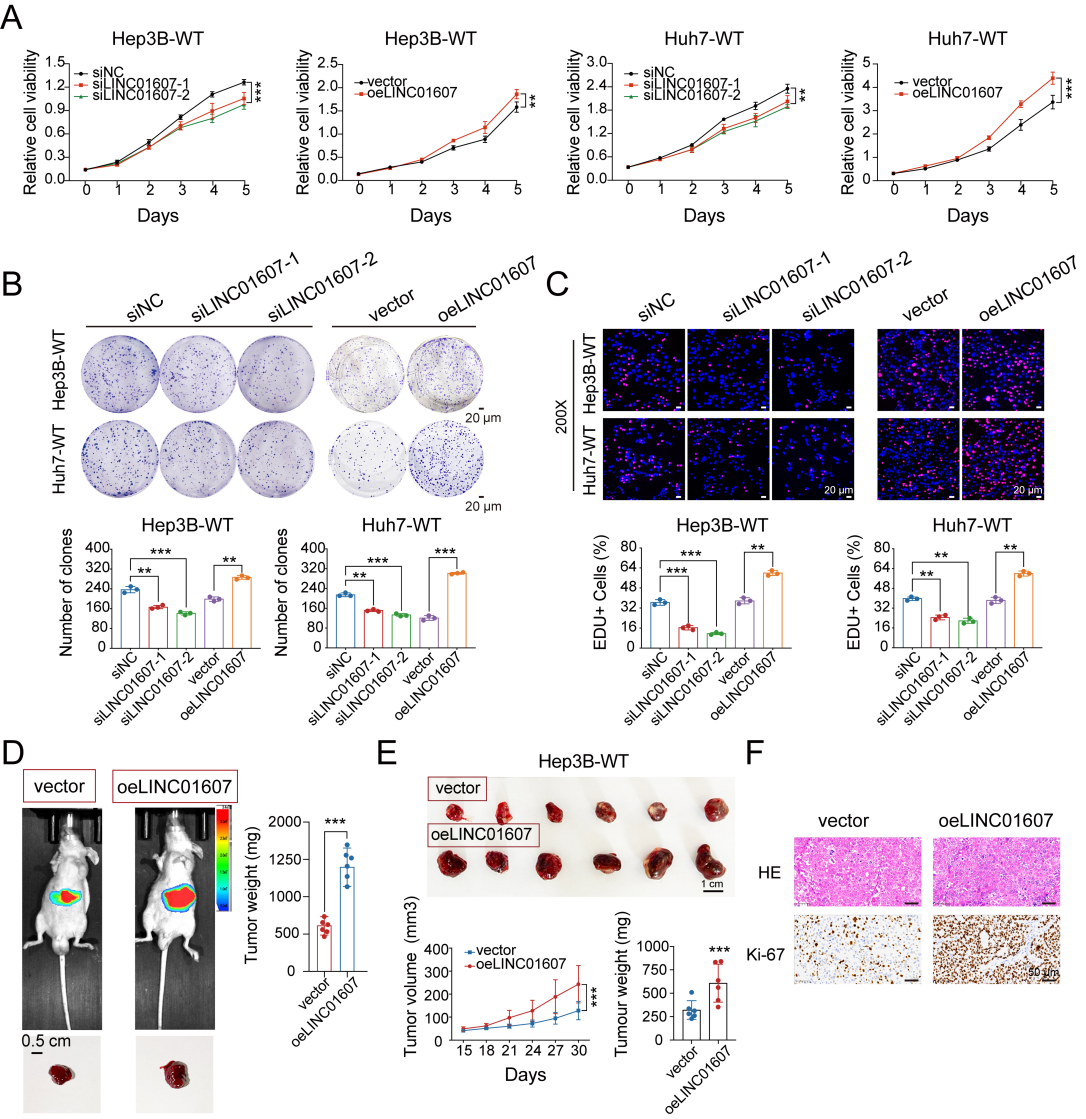
LINC01607 promotes HCC cell proliferation and tumor growth. (A-C) Cell proliferation was evaluated by CCK-8 (A), colony formation (B), and EdU incorporation (C) assays. LINC01607 overexpression enhanced proliferative capacity, while two independent siRNAs targeting LINC01607 produced the opposite effect. Quantification of colony formation and EdU-positive cells is shown below the representative images; (D) Representative bioluminescence images and gross tumor images of the HCC orthotopic model at the experimental endpoint. The heatmap depicts the photon flux emitted from tumors. Tumors were excised, and tumor weights were statistically analyzed (*n* = 6); (E) Subcutaneous xenografts were established in BALB/c nude mice using Hep3B-WT cells with LINC01607 overexpression or vector control. Tumor size was tracked during the experiment, and final tumor weights were measured at the endpoint; (F) Representative histological images of HCC tumor xenografts stained with H&E and Ki-67 IHC. LINC01607 overexpression increased tumor cellularity and Ki-67 expression, indicating enhanced proliferation. Data are presented as mean ± SD. For cell-based assays, *n* = 3 independent biological replicates unless otherwise indicated. For animal experiments, *n* = 6 mice per group. Statistical significance was assessed using two-tailed Student’s *t*-test for two-group comparisons and one-way ANOVA for multiple-group comparisons. ^**^P < 0.01, ^***^P < 0.001. HCC: Hepatocellular carcinoma; CCK-8: Cell Counting Kit-8; EdU: 5-ethynyl-2′-deoxyuridine; H&E: hematoxylin and eosin; IHC: immunohistochemistry; SD: standard deviation; ANOVA: analysis of variance.

**Figure 3:**
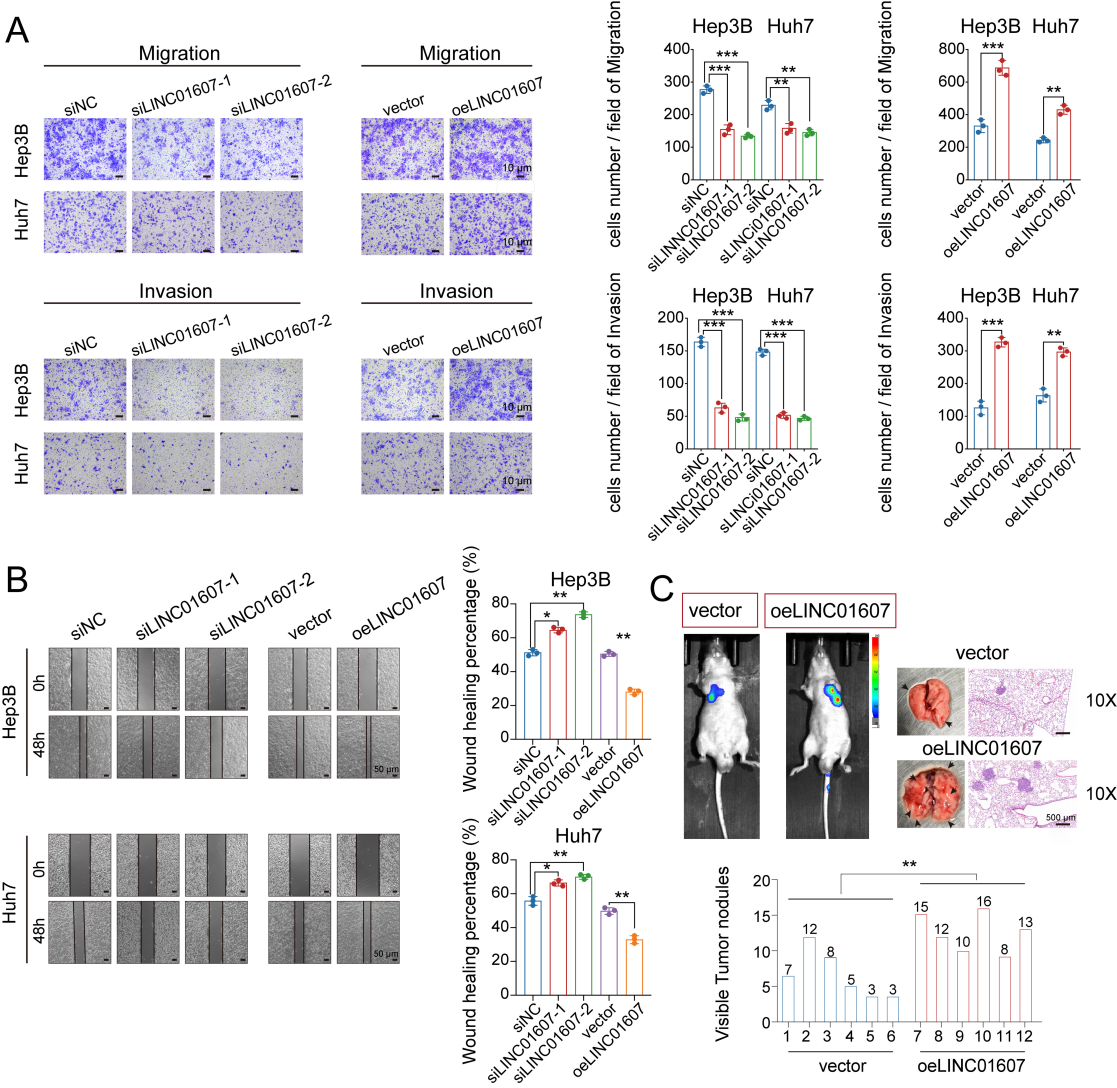
LINC01607 boosts migration and invasion of HCC cells. (A) Representative images of HCC cell migration and invasion assessed by Transwell assays after LINC01607 modulation. The quantification of migrating and invading cells is shown; (B) Wound-healing assays showed increased wound closure after LINC01607 overexpression and reduced wound closure after LINC01607 knockdown. Quantification of wound closure is shown; (C) Tail-vein injection models were used to assess lung metastasis in nude mice. Endpoint bioluminescence imaging, gross lung images, metastatic nodule counts, and H&E staining showed greater metastatic burden in the LINC01607-overexpression group. Data are presented as mean ± SD. For migration, invasion, and wound-healing assays, *n* = 3 independent biological replicates unless otherwise indicated. For the lung metastasis model, *n* = 6 mice per group. Statistical significance was assessed using two-tailed Student’s *t*-test for two-group comparisons and one-way ANOVA for multiple-group comparisons, as appropriate. ^*^P < 0.05, ^**^P < 0.01, ^***^P < 0.001. HCC: Hepatocellular carcinoma; H&E: hematoxylin and eosin; SD: standard deviation; ANOVA: analysis of variance.

To investigate the functional role of LINC01607 in HCC, we performed silencing and overexpression experiments in the Hep3B and Huh7 cell lines. Successful transfection was confirmed by qRT-PCR [Supplementary Figure 1A and B]. Cell growth was assessed using CCK-8, colony formation, and EdU assays. LINC01607 overexpression increased proliferative capacity, whereas LINC01607 silencing suppressed cell growth [Figure 2A-C]. In Transwell and wound-healing assays, cells with elevated LINC01607 displayed stronger migration and invasion, while LINC01607 knockdown weakened these phenotypes [Figure 3A and B].

In nude mouse models, stable overexpression of LINC01607 in Hep3B cells resulted in larger tumors, with significantly increased tumor volume and weight compared with control groups [Figure 2D and E]. Immunohistochemistry (IHC) revealed increased Ki-67 expression in tumors with LINC01607 overexpression [Figure 2F]. Moreover, a lung metastasis model using luciferase-labeled cells confirmed that LINC01607 overexpression markedly enhanced metastatic potential [Figure 3C]. Together, these findings suggest that LINC01607 contributes to HCC cell proliferation and metastatic capacity.

### LINC01607 depletion promotes ferroptosis-associated phenotypes in HCC

**Figure 4:**
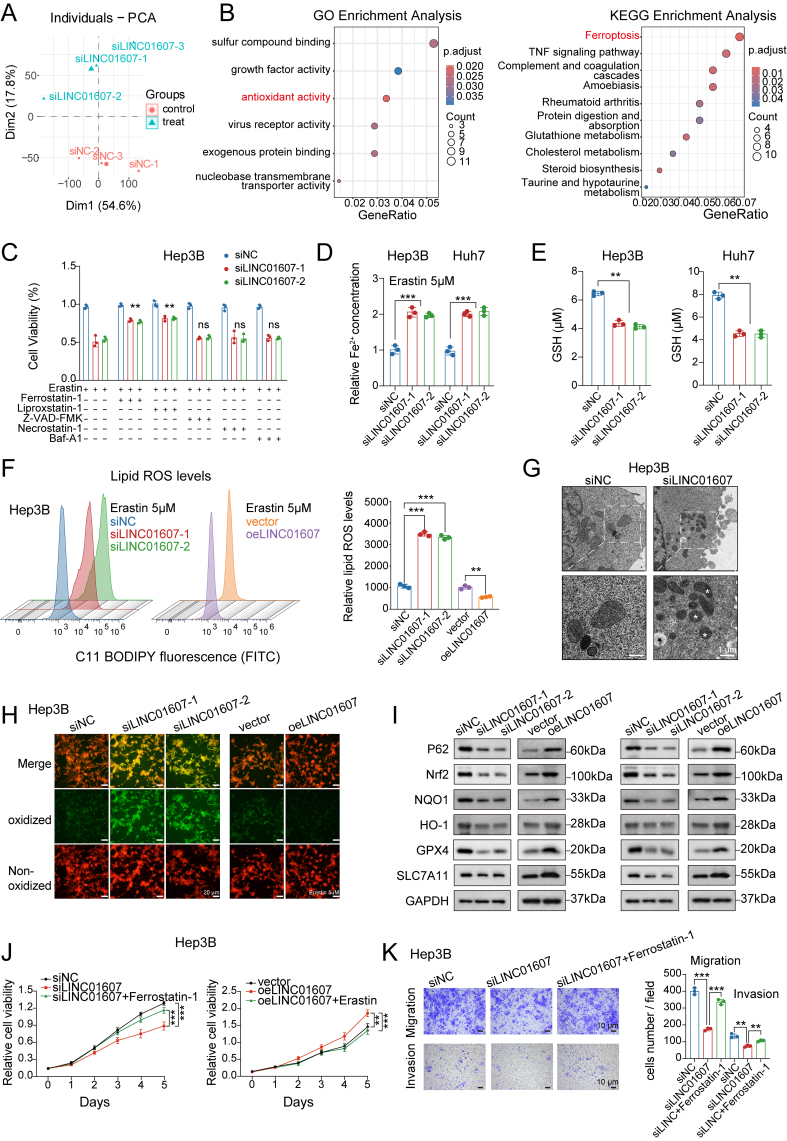
LINC01607 depletion increases ferroptosis-associated vulnerability in HCC. (A) PCA plot of RNA-seq profiles from siLINC01607- and siNC-transfected Hep3B cells. The PCA shows distinct clustering between the control and LINC01607-knockdown groups, indicating significant transcriptional changes; (B) GO and KEGG enrichment analyses were performed on differentially expressed genes and highlighted antioxidant-related functions and ferroptosis-associated pathways; (C) Cell viability assays (CCK-8) in Hep3B cells treated with ferrostatin-1 (20 μM), liproxstatin-1 (2 μM), Z-VAD-FMK (20 μM), necrostatin-1 (20 μM), or Baf-A1 (100 μM) for 24 h, followed by cotreatment with the ferroptosis inducer erastin (5 μM) for 48 h. Ferrostatin-1 and liproxstatin-1 rescued the proliferation-suppressive effects of LINC01607 knockdown, while the other inhibitors did not; (D) Intracellular Fe^2+^ levels were measured by colorimetric assay and were increased in LINC01607-silenced cells treated with erastin; (E) GSH levels were reduced after LINC01607 knockdown compared with controls; (F) Flow cytometry was used to assess intracellular lipid ROS accumulation under the indicated treatment conditions; (G) Representative TEM images of Hep3B cells demonstrated ferroptosis-associated mitochondrial shrinkage and increased membrane density in LINC01607-knockdown cells; (H) Immunofluorescence images illustrating red-to-green fluorescence shifts indicative of lipid ROS accumulation under the indicated treatments by C11 staining; (I) Western blot analysis of ferroptosis-related proteins, including SQSTM1, NRF2, NQO1, HO-1, GPX4, and SLC7A11, revealed decreased expression in LINC01607-knockdown cells and increased expression in LINC01607-overexpressing cells; (J) Ferrostatin-1 partially restored cell proliferation in LINC01607-knockdown HCC cells, as assessed by CCK-8 assays; (K) Ferrostatin-1 also attenuated the inhibitory effects of LINC01607 knockdown on migration and invasion in Transwell assays, with quantification shown. Data are presented as mean ± SD for quantitative assays. RNA-seq analysis included three independent biological replicates per group. For cell viability, intracellular Fe^2+^, GSH, lipid ROS, and rescue assays, *n* = 3 independent biological replicates unless otherwise indicated. Statistical significance was assessed using two-tailed Student’s *t*-test for two-group comparisons and one-way ANOVA for multiple-group comparisons, as appropriate. ^**^P < 0.01, ^***^P < 0.001; ns, not significant. HCC: Hepatocellular carcinoma; PCA: principal component analysis; GO: Gene Ontology; KEGG: Kyoto Encyclopedia of Genes and Genomes; CCK-8: Cell Counting Kit-8; GSH: glutathione; ROS: reactive oxygen species; TEM: transmission electron microscopy; SD: standard deviation; ANOVA: analysis of variance; TNF: tumor necrosis factor; FITC: fluorescein isothiocyanate.

To investigate transcriptional changes associated with LINC01607 depletion, we conducted RNA-seq analysis in control and LINC01607-knockdown Hep3B cells. This analysis identified 253 upregulated and 233 downregulated genes (|log2 fold change| > 1 and *P* < 0.05) [Figure 4A]. Functional enrichment analysis showed changes in antioxidant-associated biological processes and highlighted ferroptosis-related pathway enrichment in LINC01607-knockdown cells [Figure 4B].

Functional rescue assays showed that ferrostatin-1 and liproxstatin-1, but not Z-VAD-FMK, necrostatin-1, or Baf-A1, alleviated the growth-suppressive effect of LINC01607 knockdown [Figure 4C and Supplementary Figure 2A]. LINC01607 silencing also increased relative Fe^2+^ accumulation under erastin treatment compared with control cells [Figure 4D]. Therefore, we hypothesized that ferroptosis might be a significant determinant of LINC01607 knockdown-induced cell death, and we measured several ferroptotic events. We observed increased GSH depletion [Figure 4E] and lipid peroxidation [Figure 4F and Supplementary Figure 2B] upon LINC01607 knockdown. Transmission electron microscopy (TEM) of LINC01607-depleted cells revealed characteristic ferroptotic features, such as shrunken mitochondria with increased membrane density [Figure 4G]. Using C11-BODIPY staining, we observed a red-to-green fluorescence shift, which is indicative of lipid peroxidation in LINC01607-silenced cells [Figure 4H and Supplementary Figure 2C].

Ferroptosis is a complex process involving the modulation of multiple genes. To elucidate the downstream targets of LINC01607 that affect the cellular phenotype of HCC and ferroptosis, Western blot analysis showed that LINC01607 knockdown was associated with reduced expression of key ferroptosis-resistance proteins, including SQSTM1/p62, NRF2, NQO1, HO-1, GPX4, and SLC7A11. Conversely, LINC01607 overexpression increased the expression of these proteins [Figure 4I].

To determine whether ferroptosis was involved in the effects of LINC01607 depletion, ferrostatin-1 rescue experiments were performed. Ferrostatin-1 partially attenuated the inhibitory effects of LINC01607 knockdown on cell proliferation and metastatic phenotypes, as shown by CCK-8 and Transwell assays [Figure 4J and Supplementary Figure 2D; Figure 4K and Supplementary Figure 2E]. These findings suggest that LINC01607 depletion promotes a ferroptosis-associated phenotype in HCC cells.

### The p62–Keap1–Nrf2 pathway is functionally associated with LINC01607-mediated ferroptosis resistance

**Figure 5:**
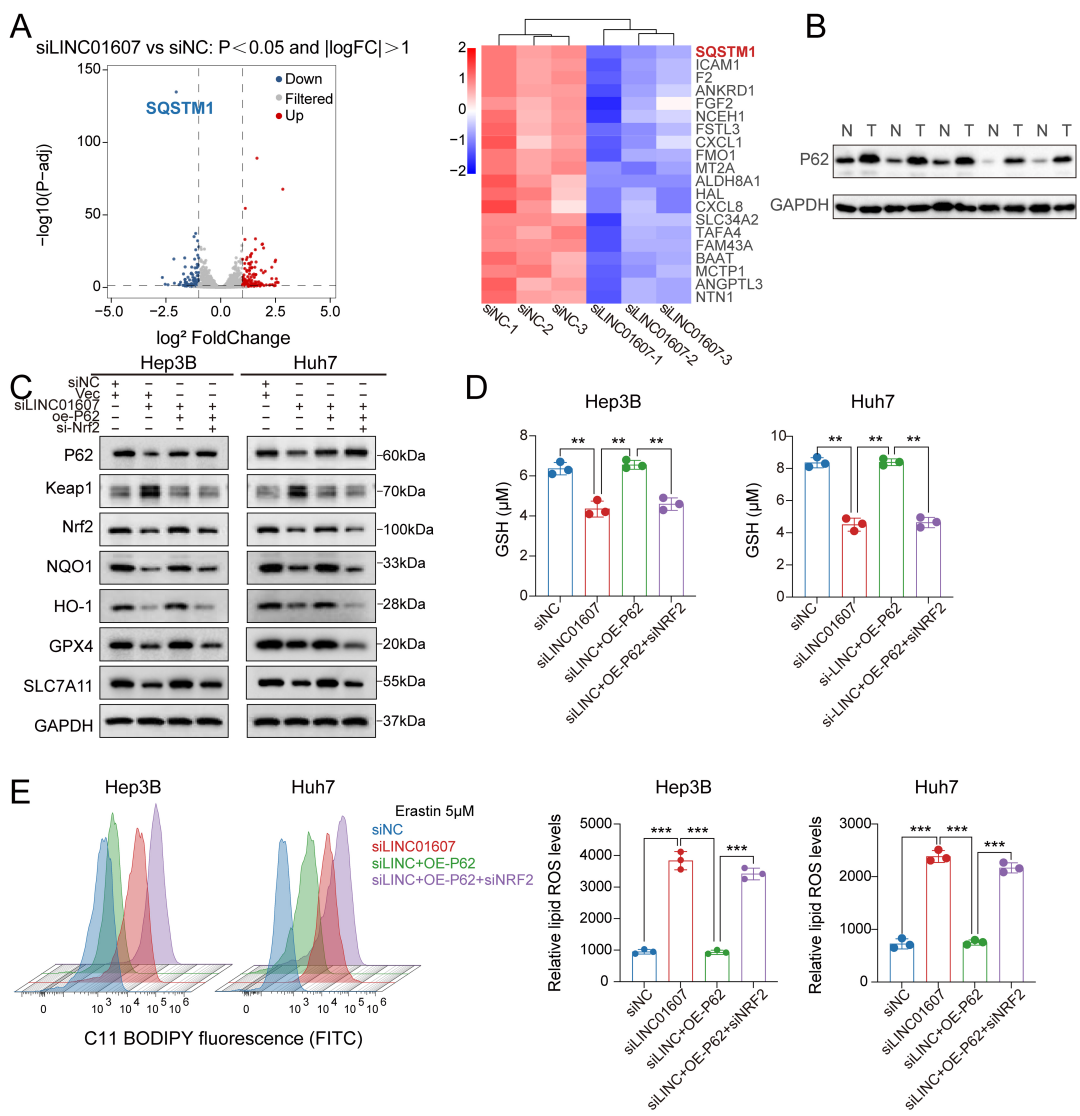
The p62–Keap1–NRF2 pathway is functionally associated with LINC01607-mediated ferroptosis resistance. (A) Volcano plot and heatmap of mRNA expression changes after LINC01607 knockdown in Hep3B cells. SQSTM1/p62 was significantly downregulated. Red, blue, and grey indicate upregulated, downregulated, and unchanged genes, respectively; (B) Western blotting showed higher p62 expression in HCC tumor tissues (T) than in matched normal tissues (N); (C) Western blot analysis in Hep3B and Huh7 cells revealed that LINC01607 knockdown was accompanied by Keap1 accumulation and reduced NRF2, NQO1, HO-1, GPX4, and SLC7A11 levels, whereas p62 overexpression partially reversed these changes. Subsequent Nrf2 silencing abrogated the rescue of the downstream antioxidant and ferroptosis-related proteins, further supporting the functional involvement of the p62–Keap1–NRF2 pathway downstream of LINC01607; (D) The decrease in GSH induced by LINC01607 knockdown was partially rescued by p62 overexpression, whereas subsequent NRF2 silencing abrogated this rescue; (E) Lipid ROS levels, measured using C11-BODIPY fluorescence, indicated that LINC01607 knockdown significantly increased lipid peroxidation. This effect was mitigated by P62 overexpression and further reversed by Nrf2 silencing. Data are presented as mean ± SD from three independent biological replicates unless otherwise indicated. Statistical significance was assessed using two-tailed Student’s *t*-test for two-group comparisons and one-way ANOVA for multiple-group comparisons, as appropriate. ^**^P < 0.01, ^***^P < 0.001. HCC: Hepatocellular carcinoma; GSH: glutathione; ROS: reactive oxygen species; SD: standard deviation; ANOVA: analysis of variance; FITC: fluorescein isothiocyanate.

To identify downstream pathways associated with LINC01607-mediated ferroptosis resistance in HCC cells, we further analyzed RNA-sequencing data from LINC01607-knockdown cells and reported that SQSTM1 (p62), a selective autophagy receptor, was among the downregulated genes [Figure 5A]. Compared with adjacent normal tissues, HCC specimens showed elevated p62 expression [Figure 5B]. SQSTM1/p62 has been reported to influence Nrf2 signaling by promoting Keap1 turnover, thereby facilitating the expression of antioxidant and ferroptosis-resistance genes such as SLC7A11, GPX4, HO-1, and NQO1^[23]^. Through this antioxidant program, Nrf2 may help limit lipid peroxidation and ferroptosis. We next overexpressed p62 in LINC01607-downregulated HCC cells. Western blot analysis confirmed that LINC01607 knockdown was accompanied by increased Keap1 levels and reduced NRF2, NQO1, HO-1, GPX4, and SLC7A11 levels, whereas p62 overexpression partially reversed these changes. Consequently, the expression of downstream antioxidant and ferroptosis resistance proteins decreased again after Nrf2 depletion [Figure 5C]. In addition, SQSTM1/p62 mRNA expression changed in parallel with p62 protein levels following LINC01607 perturbation. Consistent with the protein data, p62 overexpression reversed both the decrease in GSH levels [Figure 5D] and the increase in lipid ROS levels detected with BODIPY 581/591 C11 [Figure 5E]; both effects were reversed when Nrf2 was subsequently silenced.

These findings support a functional connection between LINC01607 and the p62–Keap1–Nrf2 pathway but do not establish a direct molecular mechanism by which LINC01607 regulates SQSTM1/p62 expression.

### LINC01607 depletion enhances sensitivity to sorafenib and ferroptosis-inducing treatment

**Figure 6:**
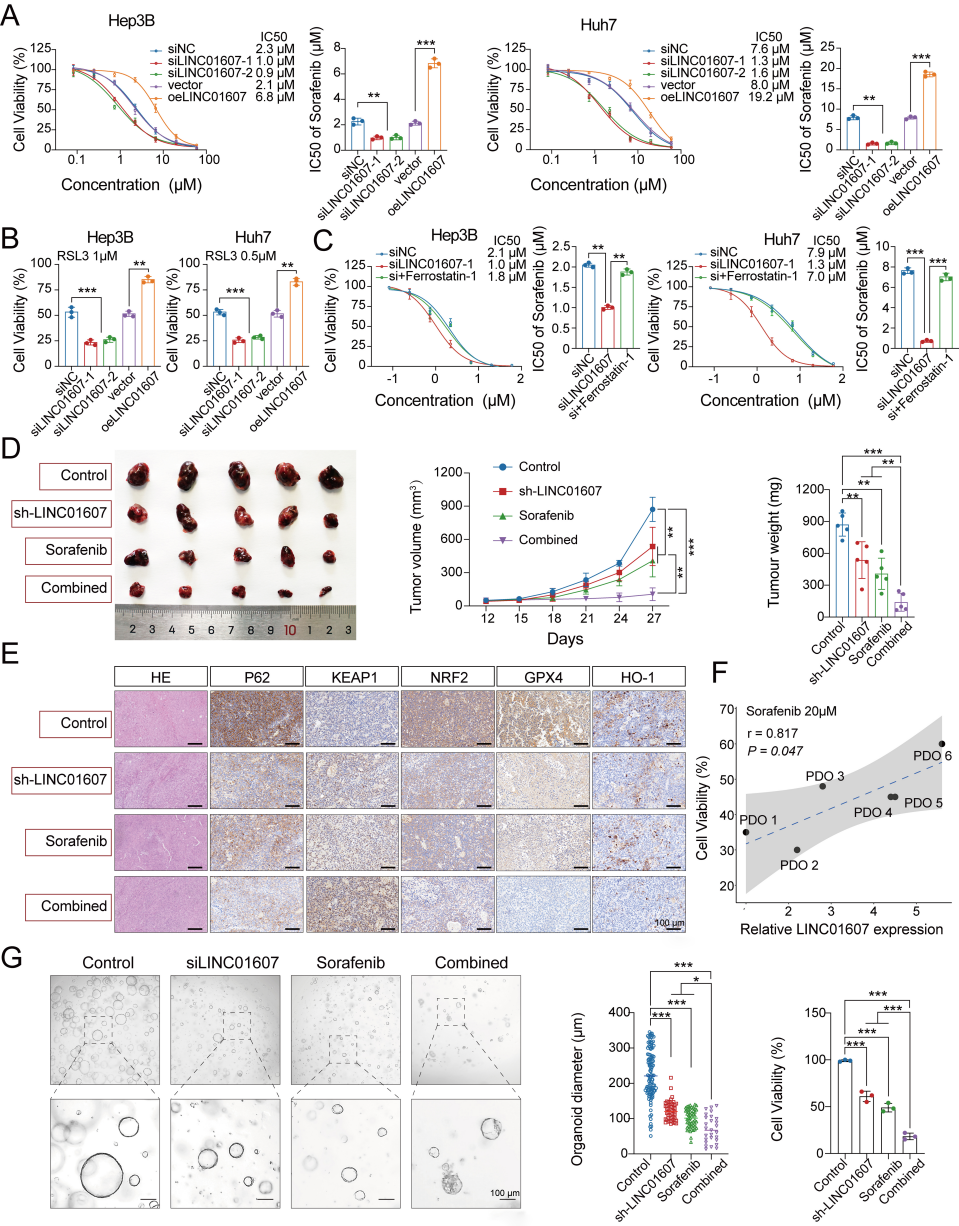
Silencing LINC01607 enhances sorafenib sensitivity in cellular, animal, and PDO models. (A) Cell viability assays (CCK-8) in Hep3B and Huh7 cells were conducted to evaluate sorafenib sensitivity under conditions of LINC01607 knockdown or overexpression. Knockdown of LINC01607 significantly reduced the IC50 of sorafenib, while overexpression increased the IC50, indicating enhanced drug resistance; (B) CCK-8 assays were used to assess RSL3 sensitivity in Hep3B and Huh7 cells with LINC01607 knockdown or overexpression; (C) Ferrostatin-1 rescue assays under sorafenib treatment were performed to determine whether ferroptosis contributed to the increased sorafenib sensitivity induced by LINC01607 knockdown; (D) Subcutaneous xenograft experiments in nude mice revealed that LINC01607 knockdown sensitized tumors to sorafenib. The combination of sh-LINC01607 and sorafenib produced a greater reduction in tumor growth and tumor weight than either treatment alone; (E) H&E and IHC staining were performed to assess the expression of p62, KEAP1, NRF2, GPX4, and HO-1 in subcutaneous xenografts; (F) CCK-8 assays were used to determine cell viability in six organoid models, and organoid viability exhibited a positive correlation with LINC01607 expression levels; (G) Bright-field images of patient-derived HCC organoids treated with sorafenib, LINC01607 silencing, or their combination for four days. LINC01607 silencing and sorafenib synergistically reduced organoid diameter and cell viability. Quantifications of organoid diameters and cell viability are shown in the right panels. Data are presented as mean ± SD. For cell-based drug-sensitivity assays, *n* = 3 independent biological replicates unless otherwise indicated. For xenograft experiments, *n* = 6 mice per group. For PDO analyses, *n* = 6 independent PDO lines for the correlation analysis, and organoid treatment assays were performed in three independent experimental replicates unless otherwise indicated. ^*^P < 0.05, ^**^P < 0.01, ^***^P < 0.001. PDO: Patient-derived organoid; CCK-8: Cell Counting Kit-8; H&E: hematoxylin and eosin; IHC: immunohistochemistry; SD: standard deviation.

Given that LINC01607 depletion promoted ferroptosis-associated phenotypes, we next asked whether this increase in ferroptosis vulnerability translated to altered drug sensitivity. We first examined the response to sorafenib, a multikinase inhibitor with ferroptosis-related activity, and then assessed the sensitivity to RSL3, a canonical ferroptosis inducer. Evaluation of sorafenib sensitivity by cell viability and IC50 analysis revealed that cells with elevated LINC01607 expression were less sensitive to sorafenib, whereas LINC01607 depletion increased sorafenib sensitivity [Figure 6A]. To test whether the effect of LINC01607 was not restricted to sorafenib alone, we next examined the response to RSL3. LINC01607 depletion significantly increased sensitivity to RSL3, as reflected by reduced cell viability. These data support that LINC01607 modulates ferroptosis-related drug sensitivity beyond sorafenib [Figure 6B]. To determine whether this phenotype was linked to ferroptosis, we performed rescue experiments under sorafenib treatment using ferrostatin-1. The ferroptosis inhibitor partially reversed the enhanced sorafenib sensitivity induced by LINC01607 depletion, indicating that ferroptosis contributes, at least in part, to this phenotype [Figure 6C]. *In vivo*, xenograft tumors derived from LINC01607-depleted cells showed an enhanced response to sorafenib [Figure 6D]. Hematoxylin and eosin (H&E) staining and IHC analysis were used to evaluate p62, KEAP1, NRF2, GPX4, and HO-1 expression in subcutaneous tumor tissues. LINC01607 knockdown and sorafenib treatment were associated with reduced p62, NRF2, GPX4, and HO-1 expression and increased KEAP1 expression. Moreover, the combination of silencing LINC01607 and sorafenib further changed the indicator levels [Figure 6E]. Under sorafenib treatment, viability of PDOs positively correlated with baseline LINC01607 expression levels across PDO lines [Figure 6F]. After LINC01607 was depleted, the organoid diameter decreased, and the cell survival rate decreased; combined treatment with sorafenib further decreased the organoid diameter and cell survival rate [Figure 6G]. Together, these findings indicate that LINC01607 depletion increases sorafenib sensitivity in association with increased ferroptosis vulnerability.

## DISCUSSION

HCC ranks among the common malignancies globally and is characterized by a high incidence rate. HCC develops through a multistep and multifactorial process, in which lncRNAs are increasingly recognized as important regulatory molecules. LncRNAs associated with HCC can influence a variety of malignant phenotypes by altering gene expression or activity^[24]^. The present study supports a functional role for LINC01607 in HCC progression and ferroptosis-associated vulnerability. Clinically, LINC01607 was upregulated in HCC tissues, and elevated LINC01607 expression was linked to poorer patient outcomes [Figure 1]. We examined 92 paired HCC tissues to analyze the correlation between LINC01607 expression and patient characteristics, the results of which are shown in Table 1. The results revealed that the LINC01607 level is correlated with tumor numbers, microvascular invasion, and TNM stage in HCC. Furthermore, our functional assays showed that LINC01607 promotes HCC cell proliferation and metastasis *in vitro* and *in vivo* [Figures 2 and 3] and confers resistance to sorafenib [Figure 6], a multikinase inhibitor with ferroptosis-related activity. These results are consistent with our prior research, which revealed that LINC01607 is an oncogene that is markedly overexpressed in lenvatinib-resistant HCC models^[8]^. These findings support a model in which LINC01607 modulates HCC progression and drug resistance, at least in part through functional modulation of the p62–Keap1–NRF2 pathway, and support further investigation of LINC01607 as a potential therapeutic target in HCC.

Importantly, the effects of LINC01607 on proliferation, metastasis, ferroptosis resistance, and drug response should not be viewed as isolated observations. Rather, our data support an integrated model in which LINC01607 enhances a tumor-intrinsic stress-adaptive state, at least in part through functional modulation of the p62–Keap1–NRF2 pathway. Within this framework, ferroptosis resistance likely serves as a central functional mediator that promotes tumor cell survival under oxidative stress and treatment pressure, thereby contributing to malignant progression and reduced sensitivity to ferroptosis-related therapy.

Compared with other types of cell death, ferroptosis has unique features in terms of cell structure, chemical processes, and genetic makeup. Disruption of ferroptosis regulatory networks contributes to HCC progression. As versatile regulators of gene expression and cellular behavior, lncRNAs may influence malignant phenotypes, in part by modulating ferroptosis-related pathways^[25,26]^. A clearer understanding of lncRNA-mediated ferroptosis regulation in HCC may provide a basis for developing new therapeutic strategies. Our results support the involvement of ferroptosis following LINC01607 depletion, as evidenced by increased lipid peroxidation, GSH depletion, enhanced Fe^2+^ accumulation under ferroptotic stress, ferroptosis-associated mitochondrial changes, and ferroptosis inhibitor-sensitive phenotypes. The reversal of these effects by ferrostatin-1 and liproxstatin-1, but not by inhibitors of apoptosis, necroptosis, or autophagy, together with the increased vulnerability of LINC01607-depleted cells to RSL3 and the partial rescue of sorafenib sensitivity by ferrostatin-1, further supports the involvement of ferroptosis in the phenotypes induced by LINC01607 knockdown [Figures 4 and 6]. These findings are consistent with previous evidence that iron-dependent lipid peroxidation contributes to ferroptosis-associated cancer cell vulnerability. By demonstrating that LINC01607 knockdown suppresses key ferroptosis resistance proteins, including SQSTM1/P62, Nrf2, SLC7A11, and GPX4, our study highlights the involvement of the p62-Keap1-Nrf2 signaling axis in modulating ferroptosis resistance [Figure 5]. This pathway is well known for its role in antioxidant defense and cancer cell survival^[27,28]^, providing a plausible explanation for the impact of LINC01607 on ferroptosis.

The identification of p62 as a downstream molecule altered by LINC01607 knockdown, together with the results of rescue experiments, supports a functional connection between LINC01607 and the p62–Keap1–Nrf2 pathway. The present data support regulation of p62 at the level of transcript abundance, although the direct molecular mechanism remains unresolved and additional post-transcriptional or protein-stability mechanisms cannot be formally excluded. Our data show that p62-mediated stabilization of Nrf2 is crucial for maintaining the expression of ferroptosis resistance genes. Overexpressing p62 in LINC01607-deficient cells rescued the expression of these genes and counteracted ferroptosis, whereas Nrf2 knockdown reversed this effect [Figure 5]. These findings are compatible with a functional role of the p62–Keap1–Nrf2 axis in oxidative stress adaptation and treatment resistance. The protective role of Nrf2 in preventing lipid peroxidation has been well documented^[29]^, and our study provides evidence that LINC01607-associated phenotypes are functionally linked to this pathway to promote HCC cell progression and ferroptosis resistance.

However, certain aspects of our findings challenge existing paradigms. While Nrf2 generally serves as a protector against oxidative damage, its activation by LINC01607 in the context of ferroptosis resistance adds complexity to the dual role of Nrf2 in cancer biology. Although Nrf2 activation is beneficial for preventing oxidative damage in normal tissues, its overactivation can facilitate cancer progression and therapeutic resistance^[30,31]^. This duality necessitates further investigation into the context-specific roles of Nrf2 and how they can be modulated therapeutically.

Our study has several limitations. Although we established the *in vitro* and *in vivo* relevance of LINC01607 in HCC progression, ferroptosis-associated phenotypes, and treatment resistance, the translational value of targeting LINC01607 remains to be defined. PDOs provide initial support for therapeutic relevance, but broader clinical validation is still needed. In addition, while our data support the functional involvement of the p62–Keap1–NRF2 pathway downstream of LINC01607, they do not fully resolve the direct molecular events linking LINC01607 to SQSTM1/p62 or the Keap1–NRF2 complex. Thus, the results of the present study support pathway involvement rather than a fully established direct mechanism. Therefore, the p62–Keap1–Nrf2 axis should be interpreted as a functionally involved downstream pathway rather than a direct linear mechanism of LINC01607 action. Similarly, although multiple lines of evidence support the involvement of ferroptosis following LINC01607 depletion, we did not perform a broader set of orthogonal validation assays. Therefore, these findings should be interpreted as evidence of ferroptosis involvement and ferroptosis-associated vulnerability rather than as proof of a fully established ferroptosis mechanism. Moreover, because sorafenib is a multikinase inhibitor with pleiotropic effects, its phenotypic consequences cannot be interpreted as exclusively ferroptosis dependent. Accordingly, our findings are best interpreted as showing that LINC01607 modulates ferroptosis-related vulnerability rather than establishing ferroptosis as the sole basis of the sorafenib response. Finally, because *in vivo* studies were conducted in BALB/c nude mice, the current work does not address the potential contribution of LINC01607 to antitumour immunity, immune evasion, or ferroptosis-associated immune remodelling. Future studies in immunocompetent or humanized models will be needed to define the role of LINC01607 within the immune microenvironment.

In summary, our findings identify LINC01607 as a functional contributor to HCC progression and ferroptosis-associated therapy resistance, with involvement of the p62–Keap1–NRF2 pathway. Targeting LINC01607 or related ferroptosis-associated pathways may represent a potential strategy to improve treatment response in HCC. Further investigation is required to define the clinical significance and therapeutic feasibility of LINC01607 inhibition in HCC.

## DECLARATIONS

### Availability of data and materials

Data in this study are available from the corresponding author based on reasonable request.

### Copyright

© The Author(s) 2026.

## References

[1] Sung H, Ferlay J, Siegel RL (2021). Global cancer statistics 2020: GLOBOCAN estimates of incidence and mortality worldwide for 36 cancers in 185 countries. CA Cancer J Clin.

[2] Bray F, Ferlay J, Soerjomataram I, Siegel RL, Torre LA, Jemal A (2018). Global cancer statistics 2018: GLOBOCAN estimates of incidence and mortality worldwide for 36 cancers in 185 countries. CA Cancer J Clin.

[3] Kudo M, Finn RS, Qin S (2018). Lenvatinib versus sorafenib in first-line treatment of patients with unresectable hepatocellular carcinoma: a randomised phase 3 non-inferiority trial. Lancet.

[4] Huarte M, Guttman M, Feldser D (2010). A large intergenic noncoding RNA induced by p53 mediates global gene repression in the p53 response. Cell.

[5] Hung T, Wang Y, Lin MF (2011). Extensive and coordinated transcription of noncoding RNAs within cell-cycle promoters. Nat Genet.

[6] Xie C, Li SY, Fang JH, Zhu Y, Yang JE (2021). Functional long non-coding RNAs in hepatocellular carcinoma. Cancer Lett.

[7] Huo X, Han S, Wu G (2017). Dysregulated long noncoding RNAs (lncRNAs) in hepatocellular carcinoma: implications for tumorigenesis, disease progression, and liver cancer stem cells. Mol Cancer.

[8] Zhang Y, Zhang Y, Tao H (2023). Targeting LINC01607 sensitizes hepatocellular carcinoma to Lenvatinib via suppressing mitophagy. Cancer Lett.

[9] Chen X, Kang R, Kroemer G, Tang D (2021). Broadening horizons: the role of ferroptosis in cancer. Nat Rev Clin Oncol.

[10] Stockwell BR, Friedmann Angeli JP, Bayir H (2017). Ferroptosis: a regulated cell death nexus linking metabolism, redox biology, and disease. Cell.

[11] Gao M, Yi J, Zhu J (2019). Role of mitochondria in ferroptosis. Mol Cell.

[12] Dixon SJ, Lemberg KM, Lamprecht MR (2012). Ferroptosis: an iron-dependent form of nonapoptotic cell death. Cell.

[13] Ajoolabady A, Tang D, Kroemer G, Ren J (2023). Ferroptosis in hepatocellular carcinoma: mechanisms and targeted therapy. Br J Cancer.

[14] Chen J, Li X, Ge C, Min J, Wang F (2022). The multifaceted role of ferroptosis in liver disease. Cell Death Differ.

[15] Li L, Xie D, Yu S (2024). WNK1 interaction with KEAP1 promotes NRF2 stabilization to enhance the oxidative stress response in hepatocellular carcinoma. Cancer Res.

[16] Zhang D, Man D, Lu J (2023). Mitochondrial TSPO promotes hepatocellular carcinoma progression through ferroptosis inhibition and immune evasion. Adv Sci.

[17] Tonelli C, Chio IIC, Tuveson DA (2018). Transcriptional regulation by Nrf2. Antioxid Redox Signal.

[18] Ma Q (2013). Role of nrf2 in oxidative stress and toxicity. Annu Rev Pharmacol Toxicol.

[19] Jaramillo MC, Zhang DD (2013). The emerging role of the Nrf2-Keap1 signaling pathway in cancer. Genes Dev.

[20] Jain A, Lamark T, Sjøttem E (2010). p62/SQSTM1 is a target gene for transcription factor NRF2 and creates a positive feedback loop by inducing antioxidant response element-driven gene transcription. J Biol Chem.

[21] Sun X, Ou Z, Chen R (2016). Activation of the p62-Keap1-NRF2 pathway protects against ferroptosis in hepatocellular carcinoma cells. Hepatology.

[22] Zhang DD (2006). Mechanistic studies of the Nrf2-Keap1 signaling pathway. Drug Metab Rev.

[23] Tian B, Lu ZN, Guo XL (2018). Regulation and role of nuclear factor-E2-related factor 2 (Nrf2) in multidrug resistance of hepatocellular carcinoma. Chem Biol Interact.

[24] Yuan D, Chen Y, Li X (2021). Long non-coding RNAs: potential biomarkers and targets for hepatocellular carcinoma therapy and diagnosis. Int J Biol Sci.

[25] Shi M, Zhang R, Lyu H (2025). Long non-coding RNAs: emerging regulators of invasion and metastasis in pancreatic cancer. J Adv Res.

[26] Xiang S, Yan W, Ren X, Feng J, Zu X (2024). Role of ferroptosis and ferroptosis-related long non'coding RNA in breast cancer. Cell Mol Biol Lett.

[27] Liu L, Zhang X, Zhang R (2023). Sohlh2 promotes pulmonary fibrosis via repression of p62/Keap1/Nrf2 mediated anti-oxidative signaling pathway. Cell Death Dis.

[28] Lee DH, Park JS, Lee YS, Bae SH (2022). PERK prevents hepatic lipotoxicity by activating the p62-ULK1 axis-mediated noncanonical KEAP1-Nrf2 pathway. Redox Biol.

[29] Yang R, Gao W, Wang Z (2024). Polyphyllin I induced ferroptosis to suppress the progression of hepatocellular carcinoma through activation of the mitochondrial dysfunction via Nrf2/HO-1/GPX4 axis. Phytomedicine.

[30] Zhou Y, Chen Y, Shi Y (2023). FAM117B promotes gastric cancer growth and drug resistance by targeting the KEAP1/NRF2 signaling pathway. J Clin Invest.

[31] Wan ZH, Jiang TY, Shi YY (2020). RPB5-mediating protein promotes cholangiocarcinoma tumorigenesis and drug resistance by competing with NRF2 for KEAP1 binding. Hepatology.

