# Supplementary material for: LINC01607 promotes hepatocellular carcinoma progression and ferroptosis-associated therapy resistance with functional involvement of the p62–Keap1–Nrf2 pathway
Source: Cancer Drug Resist. 2026 Jul 17;9:23. doi: 10.20517/cdr.2026.02 (PMC13459114; doi:10.20517/cdr.2026.02)
Supplement: Supplementary file 1 [file cdr-9-23-SupplementaryMaterials.pdf]

Supplementary Materials

LINC01607 promotes hepatocellular carcinoma progression and ferroptosis-associated therapy resistance with functional involvement of the p62–Keap1–Nrf2 pathway

Yuxin Zhang[1,2,#], Weiqi Xu[1,3,#], Fangling Cheng[4], Jinghan Zhu[1], Yuanxiang Lu[1], Guangzhen Cai[1], Jiang Li[5], Yujie Zhang[4]

[1]Hepatic Surgery Center, Tongji Hospital, Tongji Medical College, Huazhong University of Science and Technology, Wuhan 430030, Hubei, China.
[2]Department of Obstetrics and Gynecology, National Clinical Research Center for Obstetrics and Gynecology, Tongji Hospital, Tongji Medical College, Huazhong University of Science and Technology, Wuhan 430030, Hubei, China.
[3]Department of General Surgery, The First Affiliated Hospital of Nanchang University, Nanchang 330000, Jiangxi, China.
[4]Department of Oncology, Tongji Hospital, Tongji Medical College, Huazhong University of Science and Technology, Wuhan 430030, Hubei, China.
[5]Department of Hepatobiliary and Pancreatic Surgery, First Affiliated Hospital, College of Medicine, Shihezi University, Shihezi 832008, Xinjiang, China.
[#]Authors contributed equally to this work.

Correspondence to: Dr. Yujie Zhang, Department of Oncology, Tongji Hospital, Tongji Medical College, Huazhong University of Science and Technology, Wuhan 430030, Hubei, China. E-mail: tjzyj@tjh.tjmu.edu.cn; Dr. Jiang Li, Department of Hepatobiliary and Pancreatic Surgery, First Affiliated Hospital, College of Medicine, Shihezi University, Shihezi 832008, Xinjiang, China. E-mail: jiangli08@shzu.edu.cn

Supplementary Methods

Cell culture and Transfection

Hep3B (ATCC HB-8064) and Huh7 hepatocellular carcinoma cell lines were purchased from the Shanghai Institute of Cell Biology, Chinese Academy of Sciences. Hep3B cells were cultured in MEM (Gibco), whereas Huh7 cells were grown in DMEM (HyClone, UT, USA). Both media were supplemented with 10% fetal bovine serum (Gibco), and cells were maintained at 37 °C in a humidified incubator containing 5% CO_2. Full-length human SQSTM1/p62 and Nrf2 cDNAs were subsequently cloned and inserted into pcDNA3.1 vector (RiboBio) for expression. The short hairpin RNAs (shRNAs) targeting LINC01607 used for in vivo studies were synthesized and cloned into lentiviral vectors in our laboratory. According to the manufacturer's instructions, siRNA or plasmid transfection was performed using Lipofectamine™ 3000 transfection reagent (Invitrogen, USA). Cell transfection was performed according to the manufacturer's protocol. The siRNA and shRNA sequences are shown in Supplementary Table 1.

RNA extraction and quantitative reverse transcription–polymerase chain reaction

Total RNA from tissues and cell lines was extracted using TRIzol reagent (Takara, Japan) per the manufacturer's guidelines. qRT-PCR analysis was conducted with ChamQ Universal SYBR qPCR Master Mix (Vazyme Biotech Co., Ltd.) using a CFX Connect™ real-time PCR detection system (Bio-Rad, USA), following the manufacturer's instructions. GAPDH and U6 were used as endogenous reference genes. Relative lncRNA and mRNA expression was determined after normalization to the appropriate internal control and analyzed using the 2−ΔΔCt method. All assays were performed in triplicate.

Fluorescence *in situ* hybridization (FISH)

Cells were grown on coverslips in 12-well plates and subjected to FISH using a Cy3-labelled LINC01607 probe (RiboBio, China) and the Ribo™ Fluorescent In Situ Hybridization Kit (RiboBio, China), following the provided protocol. Images were acquired with a Nikon Digital ECLIPSE C1 confocal laser-scanning microscope.

Western blot

For western blotting, cells were collected and lysed in RIPA buffer supplemented with protease and phosphatase inhibitors (MedChemExpress, USA) for 30 min on ice. Total protein concentration was measured using a BCA protein assay. Equal amounts of protein lysates were subjected to 10% SDS-PAGE and transferred onto Immobilon®-P membranes (Millipore, USA). The membranes were blocked in 5% skim milk at 37 °C for 1 h and then incubated with primary antibodies overnight at 4 °C. After washing, the membranes were incubated with secondary antibodies (1:5000; Aksomics, China) at 37 °C for 1 h. Signals were developed using Bio-Rad ECL reagent and quantified with Image Lab™ 4.0 software, with GAPDH

used as the loading control. Detailed antibody information is listed in Supplementary Table 2.

Measurement of Lipid ROS

Lipid peroxidation levels in the tumours (preground and digested into single-cell suspensions) and cells were determined by staining with BODIPY™ 581/591 C11 (Invitrogen D3861) and then detection according to the manufacturer's protocol.

Cell Counting Kit 8 Assay

A cell counting kit-8 (CCK-8) assay (MedChemExpress, United States) was performed according to the manufacturer's instructions. In short, 1,000 indicated cells were seeded into 96-well plates. Cell viability was assessed using the CCK-8 assay. Briefly, at the indicated time points, cells were incubated with 100 μl of 10% CCK-8 solution at 37 °C for 2 h. The absorbance at 450 nm was measured with a microplate reader (BioTek, USA). This assay was used to evaluate cell proliferation and calculate the IC50 values of sorafenib in LINC01607-knockdown and LINC01607-overexpressing HCC cells.

EdU Incorporation Assay

For the EdU incorporation assay, HCC cells were plated in 96-well plates at a density of 4,000 cells per well and allowed to attach overnight. Cell proliferation was then assessed using the Cell-Light™ EdU Apollo567 In Vitro Kit (RiboBio, Guangzhou, China) following the manufacturer's instructions. Briefly, cells were incubated with 100 μl of 50 μM EdU working solution for 2 h, washed with PBS, fixed in 4% paraformaldehyde, and permeabilized with 0.5% Triton X-100. The incorporated EdU was visualized by staining with 100 μl of 1× Apollo reaction solution for 30 min, followed by nuclear counterstaining with 1× Hoechst 33342. Fluorescence images were acquired using a Leica fluorescence microscope, and EdU-positive cells were quantified with Image-Pro Plus 6.0.

Transwell Assay

Transwell assays were performed to evaluate the migratory and invasive abilities of HCC cells. For invasion assays, Matrigel-coated Transwell chambers (Corning, USA) were used following the supplier's instructions, while uncoated chambers were used for migration assays. A total of 6,000 cells in serum-free medium were seeded into the upper chamber, and complete medium was added to the lower chamber. Cells that traversed the membrane were fixed in 4% paraformaldehyde, stained with crystal violet, and quantified by counting five randomly chosen microscopic fields per well at ×200 magnification.

Wound Healing Assay

Cells were seeded in triplicate into 24-well plates. When the cells reached > 90% confluence, sterile micropipette tips were used to generate scratch wounds, and the medium supplemented with 10% FBS was replaced with serum-free culture medium. The scratch width was photographed under a microscope, and the

results are presented as the percent scratch closure.

C11 staining

Intracellular lipid ROS levels were detected using a C11 BODIPY 581/591 lipid peroxidation probe (Thermo Fisher, C10445, Waltham, MA, USA). Briefly, following treatment, the culture medium was removed, and cells were incubated with 10 μM Image-iT® Lipid Peroxidation Sensor (Component A; Thermo Fisher, USA) for 30 min at 37 °C. The nuclei were then stained with Hoechst dye, and cells were washed before imaging. Lipid ROS accumulation was analyzed by fluorescence microscopy.

Measurement of intracellular ferrous iron (Fe^{2+})

Intracellular ferrous iron levels were determined using the Iron Assay Kit (Colorimetric, ab83366; Abcam, Cambridge, UK) following the manufacturer's protocol. Briefly, after the indicated treatments, cells were collected and lysed in assay buffer, and the lysates were centrifuged to remove insoluble material. The resulting supernatants were incubated with the reaction mixture from the kit, and absorbance was recorded at 593 nm using a microplate reader. Fe^{2+} concentrations were quantified according to the standard curve generated with the iron standard provided in the kit and normalized to total protein concentration. Data were expressed as relative intracellular Fe^{2+} levels.

Xenograft tumour growth and metastasis assay

Male BALB/c nude mice aged four weeks were purchased from Wuhan Shulaibao Biotechnology Co., Ltd. (China) and kept under specific-pathogen-free conditions. The animal experiments were conducted in compliance with the Guide for the Care and Use of Laboratory Animals (NIH publication No. 86-23, revised 1985) and were approved by the Ethics Committee of Tongji Hospital. No license number was assigned.

For tumour growth assays, 3×10^6 Hep3B cells with LINC01607 overexpression, silencing, or control were injected subcutaneously into the flanks of nude mice (6 mice per group), with tumour volumes measured every three days and weights recorded postsacrifice. The tumour volume was calculated as $0.5 \times \text{length} \times \text{width}^2$ (mm^3).

For liver metastasis assays, 1 mm^3 tumour fragments from luciferase-labelled Hep3B cells were transplanted into the livers of anaesthetized mice. Pulmonary metastases were assessed using bioluminescence imaging, and histological analysis was performed with haematoxylin–eosin (H&E) staining.

At the experimental endpoint, the mice were euthanized by cervical dislocation, and tumour weight was measured immediately.

Construction of HCC organoid models

Organoid culture protocols were adapted from previous studies [1, 2]. Fresh HCC tissues (~0.5 cm^3) obtained shortly after surgery were rinsed in PBS, minced, and digested at 37 °C in collagenase D and

dispase II solution (2 mg/mL each) for 30–60 minutes, depending on tissue fibrosis. The digestion suspension was mixed, settled, and centrifuged at 300 × g for 5 minutes. The cell pellet was resuspended in growth factor-reduced BME and seeded (10,000 cells per well in 30 μL of BME) in a 24-well plate, overlaid with 600 μL of customized organoid culture medium. The identification of HCC organoids was carried out using H&E staining and AFP, HepPar-1 and Ki-67 immunohistochemical staining. The reagents for the organoid medium are provided in Supplementary Table 3.

RNA-seq

Total RNA was isolated from Hep3B cells transfected with siLINC01607 or negative control siRNA. Each group contained three independent biological replicates, referred to as siNC-1, siNC-2, siNC-3 and siLINC01607-1, siLINC01607-2, siLINC01607-3. RNA quantity was measured with a NanoDrop 2000 spectrophotometer (Thermo Scientific, USA), while RNA integrity and quality were assessed using an Agilent 2200 system (Agilent, USA). RNA sequencing libraries were prepared with the Ion Proton Total RNA-Seq Kit v2 following the manufacturer's protocol (Life Technologies, USA), and sequencing was conducted by BGI Biotech Company (Shenzhen, China).

Supplementary Table 1. siRNA and shRNA sequence used in this study.

Name	Sequence	Company
LINC01607-siRNA-1	CGACCATGAATCCAGACAT	RiboBio
LINC01607-siRNA-2	CCATCGTCACTCTTACCAA	RiboBio
LINC01607-shRNA	CCGGACCATCGTCACTCTTACCAATCTCGAGATTGGT<br>AAGAGTGACGATGGTTTTTT	Vigene<br>Biology
Nrf2-siRNA	CCAAAGCTAGTATAGCAATAA	RiboBio

Reagents and antibodies

Ferrostatin-1 (S7243), Liproxstatin-1(S7699), Z-VAD-FMK (S7023), Necrostatin-1 (S8037), Baf-A1(S1413), Erastin (S7242) were obtained from Selleck.

Supplementary Table 2. Antibodies used in this study

Name	Company	Catalog Number	Assay
SQSTM1/p62	Cell Signaling Technology	#23214	WB (1:1000), IHC (1:200)
GAPDH	Proteintech	60004-1-lg	WB (1:10000)
Ki-67	Proteintech	27309-1-AP	IHC (1:5000)
Nrf2	Proteintech	16396-1-AP	WB (1:4000), IHC (1:200)
NQO1	Proteintech	11451-1-AP	WB (1:4000)
HO-1	Proteintech	10701-1-AP	WB (1:3000), IHC (1:200)
GPX4	Proteintech	67763-1-lg	WB (1:2000), IHC (1:1000)
SLC7A11	Proteintech	26864-1-AP	WB (1:2000)
KEAP1	Proteintech	10503-2-AP	WB (1:2000), IHC (1:200)

Supplementary Table 3. Reagents for organoid medium

Name	Company	Catalog Number
B27	Gibco	12587010
N2	Gibco	17502048
N-acetyl-l-cysteine	Sigma	A9165
L-GlutMax	Gibco	35050-061
HEPES	Gibco	15630106
r-hEGF	Peprotech	AF-100-15-500
r-hFGF-10	Peprotech	100-26-1000
r-hHGF	Peprotech	100-39-1000
R-Spondin1	Peprotech	120-38-1000
Wnt3a	R&D	5036-WN-500
TGFa	Peprotech	100-16A-500
Forskolin	Torcis	1099
A8301	Torcis	2939
Y27632	Stemcell	72304
Accutase	Stemcell	07922
Collagenase D	Roche	11088882001
Dispase II	Roche	04942078001
BME	R&D	3533-010

Results

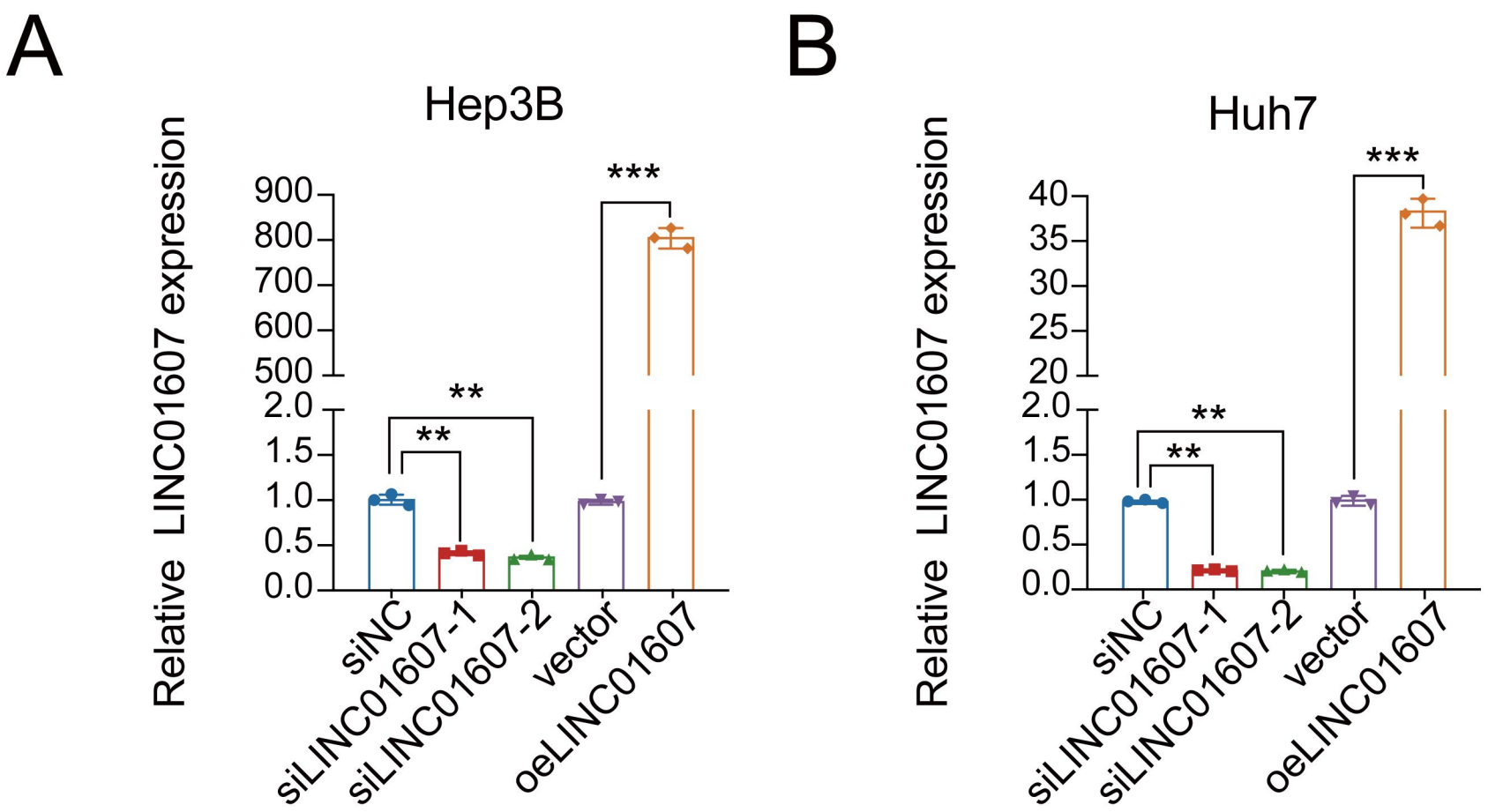

Supplementary Figure 1. RT–qPCR analysis of LINC01607 knockdown or overexpression efficiency in Hep3B (A) and Huh7 (B) cells. Data are presented as mean ± SD. n = 3 (three independent biological replicates). **P < 0.01, ***P < 0.001, **P < 0.0001 vs. control.

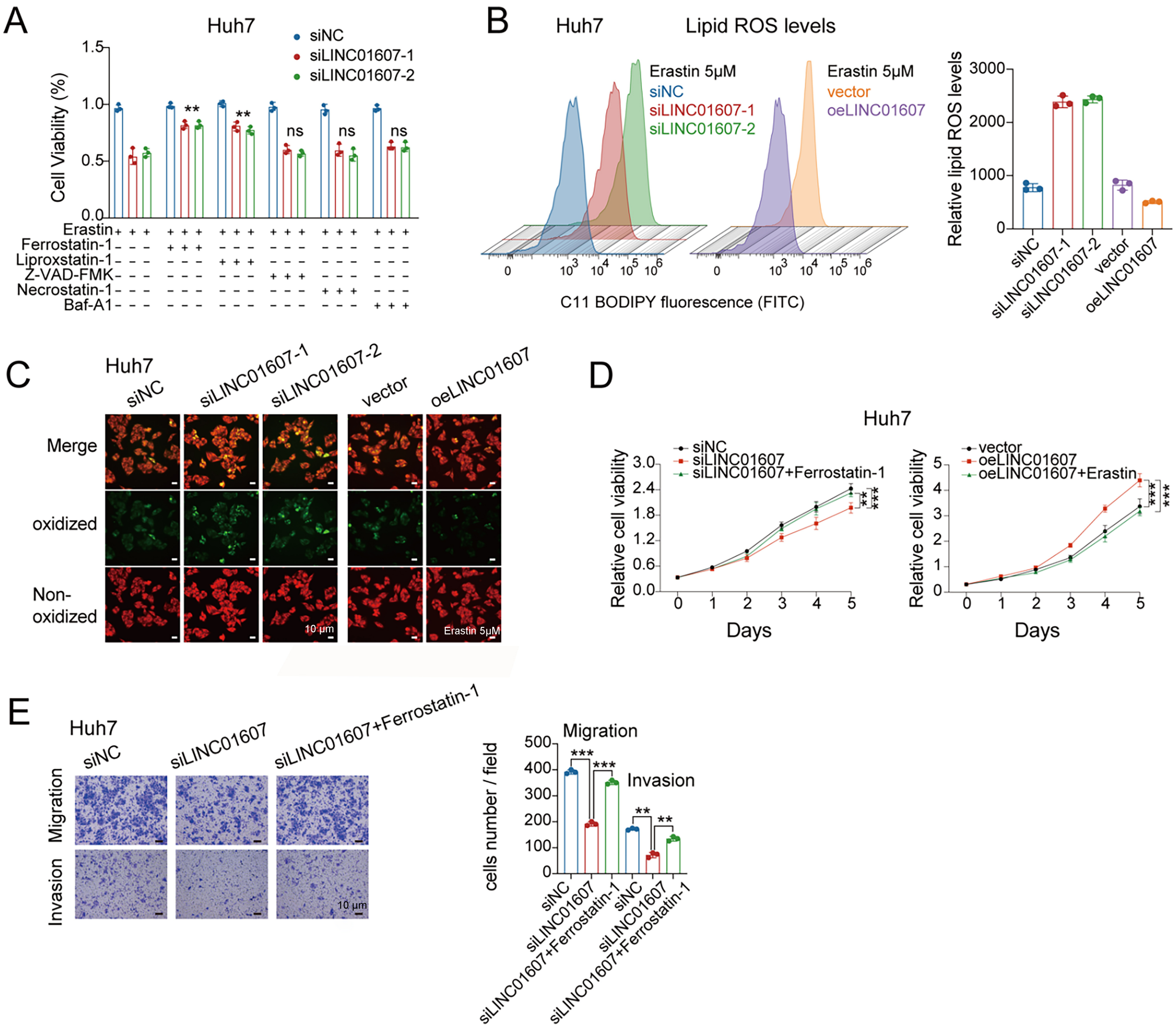

Supplementary Figure 2. Reduction of LINC01607 enhances ferroptosis and suppresses cell progression in HCC. (A) CCK-8 assays in Huh7 cells treated with ferrostatin-1 (20 μM), liproxstatin-1 (2 μM), Z-VAD-FMK (20 μM), necrostatin-1 (20 μM), or Baf-A1 (100 μM) for 24 h, followed by co-treatment with the ferroptosis inducer erastin (5 μM) for 48 h. Ferrostatin-1 and liproxstatin-1, but not the other inhibitors, rescued the proliferation-suppressive effect induced by LINC01607 knockdown. (B) Intracellular lipid ROS levels were measured by flow cytometry under the indicated treatments. (C) Immunofluorescence images illustrating red-to-green fluorescence shifts indicative of lipid ROS accumulation under the indicated treatments. (D) CCK-8 assays showed that ferrostatin-1 rescued the proliferation defect caused by LINC01607 knockdown in Huh7 cells. (E) Transwell assays showed that ferrostatin-1 mitigated the suppression of migration and invasion in LINC01607-knockdown Huh7 cells. Data are presented as mean ± SD. n = 3 (three independent biological replicates). $^{**}P < 0.01$, $^{***}P < 0.001$, $^{****}P < 0.0001$ vs. control.
